# LCM-RNAseq Highlights Intratumor Heterogeneity and a lncRNA Signature from Archival Tissues of GH-Secreting PitNETs

**DOI:** 10.3390/genes15111426

**Published:** 2024-10-31

**Authors:** Luca Cis, Simona Nanni, Marco Gessi, Antonio Bianchi, Sara De Martino, Valeria Pecci, Davide Bonvissuto, Angela Carlino, Luciano Giacò, Guido Rindi, Claudio Sette, Claudio Grassi, Carlo Gaetano, Alfredo Pontecorvi, Antonella Farsetti

**Affiliations:** 1 Department of Translational Medicine and Surgery, Università Cattolica del Sacro Cuore, 00168 Rome, Italy; luca.cis@unicatt.it (L.C.); antonio.bianchi1@unicatt.it (A.B.); valeria.pecci@unicatt.it (V.P.); alfredo.pontecorvi@unicatt.it (A.P.); 2 Fondazione Policlinico Universitario A. Gemelli IRCCS, 00168 Rome, Italy; ange.carlino@gmail.com (A.C.); claudio.sette@unicatt.it (C.S.); claudio.grassi@unicatt.it (C.G.); 3 Department of Woman and Child Health Sciences and Public Health, Fondazione Policlinico Universitario A. Gemelli IRCCS, 00168 Rome, Italy; marco.gessi@policlinicogemelli.it (M.G.); guido.rindi@unicatt.it (G.R.); 4 ENETS Center of Excellence for the Diagnosis and Cure of Neuroendocrine Tumors, 00168 Rome, Italy; 5 National Research Council (CNR)–Istituto di Analisi dei Sistemi ed Informatica “Antonio Ruberti” (IASI), 00185 Rome, Italy; sara.demartino@iasi.cnr.it (S.D.M.); antonella.farsetti@cnr.it (A.F.); 6 Department of Neuroscience, Università Cattolica del Sacro Cuore, 00168 Rome, Italy; davide.bonvissuto@unicatt.it; 7 Bioinformatics Core Facility, Gemelli Science and Technology Park (G-STeP), Fondazione Policlinico Universitario A. Gemelli IRCCS, 00168 Rome, Italy; luciano.giaco@policlinicogemelli.it; 8 Section of Pathology, Department of Life Sciences, Università Cattolica del Sacro Cuore, 00168 Rome, Italy; 9 Laboratory of Epigenetics, Istituti Clinici Scientifici Maugeri IRCCS, 20138 Pavia, Italy; carlo.gaetano@icsmaugeri.it

**Keywords:** GH-secreting pituitary neuroendocrine tumor, laser capture microdissection, precision medicine, RNA sequencing, biomarkers

## Abstract

Background: This study explores the potential for hidden variations within seemingly uniform regions of growth hormone-secreting pituitary neuroendocrine tumors (GH-PitNETs). We employed archived tissue samples using Laser Capture Microdissection Sequencing (LCM-RNAseq) to probe the molecular landscape of these tumors at a deeper level. Methods: A customized protocol was developed to extract, process, and sequence small amounts of RNA from formalin-fixed, paraffin-embedded (FFPE) tissues derived from five patients with GH-secreting PitNETs and long-term follow-up (≥10 years). This approach ensured precise isolation of starting material of enough quality for subsequent sequencing. Results: The LCM-RNAseq analysis revealed a surprising level of diversity within seemingly homogeneous tumor regions. Interestingly, the 30 most highly expressed genes included the well-known long noncoding RNA (lncRNA) MALAT1. We further validated the levels of MALAT1 and of other tumor-associated lncRNAs using digital droplet PCR. Conclusions: This study demonstrates the potential of LCM-RNAseq to unlock hidden molecular diversity within archived pituitary tumor samples. By focusing on specific cell populations, we identified lncRNAs expressed at different levels within the tumors, potentially offering new insights into the complex biology of GH-secreting PitNETs. This evidence prompts further research into the role of lncRNAs in pituitary neuroendocrine tumor aggressiveness and personalized treatment strategies.

## 1. Introduction

The anterior pituitary gland plays a pivotal role in the endocrine system. It comprises a diverse array of cells, including six primary hormone-secreting types: prolactin (PRL), growth hormone (GH), adrenocorticotrophic hormone (ACTH), thyrotrophic hormone (TSH), follicle-stimulating hormone (FSH), and luteinizing hormone (LH). The hormones secreted by this gland control multiple body processes, such as growth, cell metabolism, and reproduction. The gland also hosts other non-secretory cells, such as follicular-stellate and endothelial cells, contributing to its structural integrity and overall functionality [1,2].

Pituitary neuroendocrine tumors (PitNETs) affect approximately 5% of people globally and pose significant challenges in neuroendocrinology [3,4,5]. They are a prevalent cause of brain tumors, distinguished by their hormonal overexpression profiles, which include the most common lactotroph and the rarer thyrotrophic tumors. The clinical manifestations of these tumors can be significant, with somatotroph adenomas leading to acromegaly in adults and gigantism in children due to excess growth hormone (GH) and insulin-like growth factor 1 (IGF-1) production [4]. These tumors represent 15–20% of all PitNETs occurring as sporadic contexts in 95% of cases [6]. Increasing rates of acromegaly have been detected in the past decade, especially in areas of high industrial pollution, with a slight predominance of female individuals [7,8].

As common functional PitNETs, somatotroph adenomas arise from PIT1 lineage cells and cause acromegaly due to excessive GH and IGF-1 levels. High GH/IGF-1 serum levels lead to comorbidities, including arthritis, facial changes, prognathism, and glucose intolerance [5,9]. The treatment landscape for PitNETs typically involves surgery, which is successful in about 70% of cases. However, the effectiveness of pharmacological treatments, such as somatostatin analogs (SSAs), varies significantly among patients, highlighting the need for more detailed molecular insights to guide therapy choices [10,11,12]. Unfortunately, the factors determining a patient’s positive or negative response to treatment with SSAs, such as pasireotide and temozolomide, remain unclear [10,11,12]. Despite advancements in histopathological categorization, the molecular basis of GH PitNETs remains inadequately understood, partly due to the scarcity of preclinical models and surgical specimens available for research [13].

In the last few years, a more profound molecular characterization of PitNETs has revolutionized this field of research thanks to several molecular biology studies and omics approaches, strengthening the prediction capacity of better understanding patients’ responsiveness to pharmacological and/or surgical treatments [3,14,15]. In addition, recent developments in molecular biology, particularly those involving noncoding RNAs (ncRNAs), such as microRNAs (miRNAs) and long noncoding RNAs (lncRNAs), have provided new perspectives on the pathogenesis of PitNETs. These ncRNAs play crucial roles in gene regulation, influencing cellular processes like proliferation and apoptosis, which are central to tumor development and progression. Their study has begun to unravel the complex molecular interactions underlying PitNETs, offering potential for novel diagnostic and therapeutic strategies [3,16,17].

Recent studies have proposed that RNAs can regulate the progression of pituitary tumors, including somatotropinomas, by altering the expression of genes involved in cell proliferation and apoptosis, among other processes. Several ncRNA transcripts have been reported to present dysregulation in PitNETs. Among lncRNAs, there are MEG3, H19, GAS5, LINC00473, and SNHG12, whereas among miRNAs, the following have been reported: miR-183, miR-212, miR-16 and miR-34a, as well as circNFIX circular RNAs [18,19,20,21]. In addition, somatotropinomas exhibit frequent variation in the expression of the following mRNAs: T-PIT, NF1, NR5A1 (SF1), PIT-1, PRKARIA, GPR101, DRD2, miRNAs associated with the long noncoding RNA MEG3 and miR532-let7 (miR-574, miR-195, miR-497-5p e let-7) cluster, and lncRNAs such as H19 and MEG3 that are differentially expressed as compared to normal tissue. Recurrent alterations in the expression of coding genes, such as MEN1, MGMT, AIP, GNAS, USP48, and USP8, have also been found in other PitNET subtypes [3,16,20,22]. However, the specific role of noncoding RNAs in pituitary tumorigenesis and their potential use as therapeutic targets or predictive biomarkers for the treatment response in pituitary adenomas, including somatotropinomas, remain to be fully elucidated.

This introduction underscores the significance of advancing our understanding of the molecular characteristics of GH-secreting PitNETs. Leveraging cutting-edge techniques, such as Laser Capture Microscopy (LCM) from formalin-fixed paraffin-embedded (FFPE) tissues coupled with high-throughput RNA sequencing analysis, can help identify new biomarkers and therapeutic targets. Such efforts promise to refine precision medicine approaches for treating PitNETs, potentially leading to more effective and targeted interventions. By exploring the molecular landscape of these tumors, mainly through the analysis of ncRNA expression, we might provide novel information helpful in neuroendocrinology diagnosing and managing one of the most common intracranial tumors.

## 2. Methods

### 2.1. Patients’ Cohort

Five patients (designed A–E) with a diagnosis of primary sporadic GH-secreting PitNET who underwent transsphenoidal surgery for tumor removal at the Department of Translational Medicine and Surgery, Fondazione Policlinico Universitario A. Gemelli IRCCS-Università Cattolica, Rome, Italy (from January 2010 to December 2013) were included in the study (Table S1). All samples were diagnosed according to the World Health Organization classification system. Immunohistochemical staining was performed on each sample for the following markers: GH, Synaptophysin, Cytokeratin CAM 5.2, Ki67, and p53 (Figure S1). Two patients (A and E) exhibited residual tumor regrowth, whereas patients B, C, and D went into remission following neurosurgery (observation time: ≥10 years post-surgery). For the validation by digital droplet PCR (ddPCR) of the lncRNA transcripts, an independent group of PitNET patients (N. 6; F/M = 4/2; age (mean, min-max = 46.83 years, 24–58 years) with clinical–pathological features similar to the original cohort was included.

### 2.2. Sample Collection and Preparation

FFPE blocks were stored at room temperature and protected from light. Regions of interest (ROI) were isolated from freshly prepared 10 microns FFPE sections using the LDM6 Laser Capture Microdissector (Leica Microsystems, Wetzlar, Germany). Microdissected samples were directly placed into RNase-free tubes. Three to four small ROI of approximately 14,000 µm^2^ each were selected from each slide to obtain about 50 cells after laser microdissection (Figure S2). As a reference for the FFPE sample, two regions were microdissected and processed together (Pit 29, see Table S2). RNA extraction was performed according to the manufacturer’s instructions under RNase-free conditions (Mechery Nagel, NUCLEO SPIN total RNA FFPE). QFX fluorometer (Denovix) was used for yield analysis (RNA concentration between 30 and 120 pg/µL). The distribution value 200 (DV200) quality metric was determined using a 2100 Bioanalyzer (Agilent); four samples were DV200 > 60% (Pit 4, 11, 34, 36), and six samples <60% (Pit 1, 3, 6, 35, 18, 29). Library preparation was performed using SMARTSEQ STRANDED KIT (#634442, Takara Bio, Kusatsu, Shiga, Japan) as indicated in the user guide. Ten libraries were prepared from 200 to 800 pg of DNase-treated total RNA per FFPE sample with/without fragmentation step, according to DV200 > 60% or <60%, respectively. The ranges of libraries concentration lay between 3.6 and 17.9 nM (mean length 390 bp). Libraries were paired-end sequenced on NovaSeq 6000 (Illumina, San Diego, CA, USA) with a read length of 2 × 100 base pairs according to the manufacturer’s instructions.

### 2.3. Preprocessing of RNA Sequencing Data

The raw binary base call (BCL) output produced by the sequencer was transformed into the FASTQ format using Illumina’s bcl2fastq tool using the “dummy” sample sheet provided by Cogent NGS Analysis Pipeline software (Version 1.3.0; 2019 Illumina, Inc.) to generate the unique undetermined sample fastq file. In the subsequent step, the ‘demux’ procedure of the Cogent pipeline (Version 1.5; 2021 Takara Bio USA, Inc., San Jose, CA, USA) was used to generate individual FASTQ files for the samples. The FASTQC software Version 0.11.9 [23] was utilized to assess the per-base quality of the sequences, the GC content, and the per-sequence duplication level (https://www.bioinformatics.babraham.ac.uk/projects/fastqc/, accessed on 2 February 2024) [23]. RNA-Seq data were analyzed using the Cogent NGS Analysis Pipeline software. This procedure involved trimming the reads via Cutadapt Version 4.1 [24], aligning the sequences using STAR Version 2.7 [25] with the reference genome (hg19), calculating unique start and stop positions (Unique Start Stop, USS) via SAMtools Version 1.16.1 [26], and quantifying gene expression using Subread Version 2.0.1 [27]. The pipeline delivered a Gene Expression Matrix and a sequencing details statistics file in comma-separated values (CSV) format. The Gene Expression Matrix file included gene names as rows and patient IDs as columns, while the statistics file contained information such as used barcodes, sample names, categories of mapped and unmapped reads, gene count, and strand specificity.

### 2.4. Quality Assessment

The count matrix was imported into the RStudio environment Version 4.2.1 [28], and a subset of this matrix was generated by selecting the genes that had at least one read mapped on a minimum of one sample. Data from all ten sequenced libraries were incorporated during this initial analysis stage. The count matrix, a product of data preprocessing, comprised 10 sample barcodes as columns and 57,905 analyzed genes as rows. The exploratory analysis was conducted regardless of the presence or absence of recurrence. An initial filter reduced the matrix to 9 columns and 57,901 genes by excluding the Pit 6 (patient A) sample due to its low average number of mapped reads. Additionally, specific genes such as ENSG00000272060_RNA18S5, ENSG00000266658_RNA28S5, ENSG00000211459_MT-RNR1, and ENSG00000210082 were removed, as they should have been eliminated during the laboratory RNA purification step. Genes that had no globally mapped reads were also excluded from the analysis. Subsequently, genes with ten or more reads were selected for each sample individually. The number of reads mapped per gene was visualized through a bar plot (Figure S3).

### 2.5. Data Transformation and Visualization

For subsequent analyses, we used the DESeq2 R package (https://doi.org/10.1186/s13059-014-0550-8) [29], the data were normalized using the normTransform function to calculate the log2(x + 1) transform of the DESeqDataSeq object. The results were visualized by generating a heatmap of the count matrix for the top thirty most expressed genes and a heatmap depicting sample-to-sample distances (heatmap, ver.1.0.12). A principal component analysis (PCA) was also conducted using the plot PCA function.

### 2.6. Gene Expression Analysis by Droplet Digital PCR (ddPCR)

According to the manufacturer’s instructions, RNA extraction from FFPE tissues and cDNA preparation were performed using the NucleoSpin total RNA FFPE XS kit (Macherey-Nagel, Düren, Germany) and high-capacity kit (Applied Biosystems, Waltham, MA, USA). cDNA preamplification and ddPCR were as described in [30]. Briefly, 1 μL of cDNA was used in the preAmp step, and 2 μL of preAmp (1:100–1:1000 dilution) was used to perform ddPCR on QX200 instrument (Biorad, Hercules, CA, USA) using Eva Green (total droplet number > 10,000). Primers for MALAT1, NEAT1, H19, MEG3, and the control genes P0 and GAPDH were as [30]. Gene quantification was expressed in copy number/microliter. LncRNA level was normalized to housekeeping genes P0 and/or GAPDH.

## 3. Results

Predicting individual recurrence risk in PitNETs is crucial for a personalized medicine approach. However, current methods based on limited clinical–pathological elements are insufficient. A recent single-cell RNA sequencing (RNA-seq) study has offered promising insights [15], but its validation remains necessary. This study tackles this gap by utilizing Laser Capture Microscopy (LCM) coupled with RNA-seq on archived paraffined samples. By analyzing specific cell populations within tumors, we aim to identify precise molecular markers that accurately predict recurrence risk, paving the way for personalized management strategies for PitNET patients.

### 3.1. Transcriptomic Profile from FFPE GH-Secreting-PitNET by LCM-RNA-Seq

Five patients diagnosed with GH-secreting-PitNET (designed A-E) were included in the study, and FFPE-derived LCM samples were subjected to RNA-seq (Tables S1 and S2). Two patients (A and E) exhibited residual tumor regrowth (defined as Recurrent), whereas patients B, C, and D recovered following neurosurgery (observation time: ≥10 years post-surgery). FFPE sections were selected based on a check quality by the pathologist (Figure S4) on the H&E staining. Selected slides were subjected to LCM to obtain several areas from the same histological slide containing approximately 50 cells each after cutting. RNA was then extracted from the microdissected samples and processed for sequencing. Ten libraries were obtained and included in the bioinformatic analysis (Table S2). By setting ten mapped reads/genes as a threshold, we evaluated the expression of the single samples through bar plots (Figure S3). Pit 6 was excluded from the bioinformatics analysis with this filter. Overall, we obtained a restricted transcriptional profile of about 300 genes/sample ranging from 50 to 600 genes/sample. Sample Pit29, derived from 2 LCM areas collected, was used as a reference sample, resulting in 2561 total genes (Table S2). Next, as summarized in Table S3, we tried to establish if genes were common to the samples derived from the same FFPE slide. We noticed that samples from patient E exhibited a considerable number of shared genes despite the very different percentage of reads mapped between the two extracted samples (83 and 2561, respectively). On the other hand, specimens from patients A and C showed fewer genes in common, although the numbers of mapped reads were not that far apart (306 and 150 for A, 413, 150, and 683 for C).

**Figure 1:**
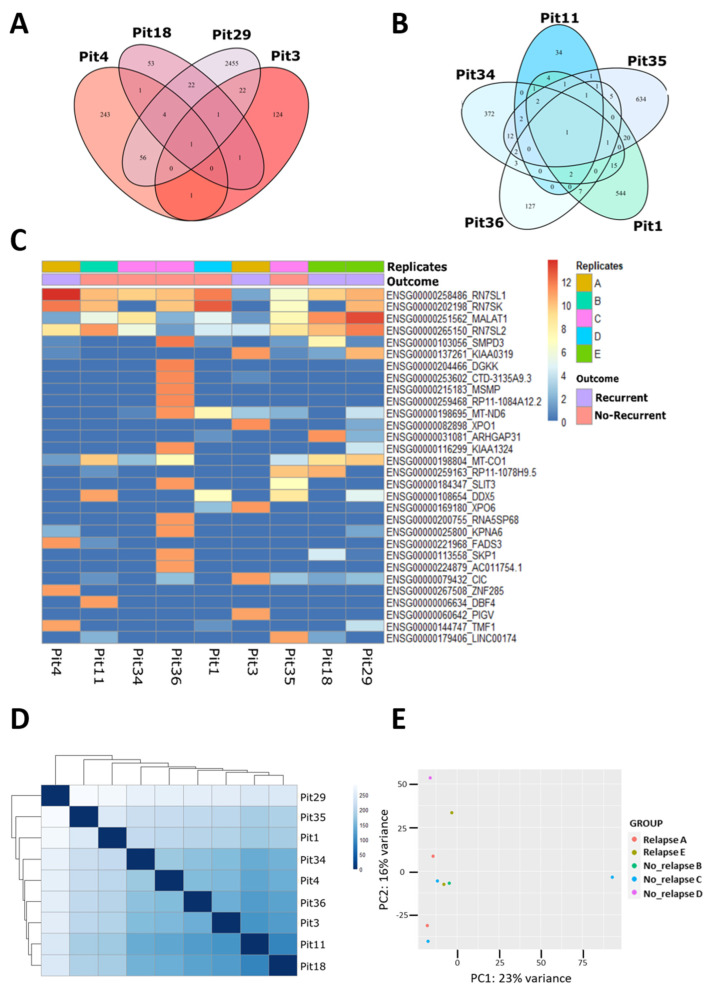
Transcriptome analysis by Laser-Capture Microdissection (LCM)-RNAseq of Growth Hormone (GH)-secreting PitNET. (**A**,**B**) Venn diagram of the genes expressed in common by the Recurrent (left) and Non-Recurrent (right) GH-secreting PitNET, respectively. (**C**) Heatmap of the genes with higher (red) or lower (blue) expression. Each sample is identified by its name and grouped according to the outcome and the patient from which it was derived. (**D**) Heatmap of the sample-to-sample distance: compares sample-sample pairs, displaying the greater or lesser difference between them on a blue scale, from lightest to darkest. (**E**) PCA of the samples, according to the groups of interest, “Outcome” (Recurrent/Non-Recurrent) and “Replicates” (**A** = Pit4 and Pit3, **B** = Pit11, **C** = Pit34, Pit35 and Pit36, **D** = Pit1, **E** = Pit18 and Pit29) on the normalized data of the whole dds (DESeqDataSet).

We used Venn diagrams to investigate gene expression potentially associated with the specific outcome (Figure 1A,B). The diagrams associated with the recurrent (left) or non-recurrent (right) conditions showed a very low number of shared genes expressed among samples. We found that all the recurrent tumor samples shared only the RN7SL1 gene, whereas those non-recurrent only the RN7SL2 (see also Table S3), which encode for two subunits of the cytoplasmic complex named RNA component of signal recognition particles, stressing the intrinsic heterogeneity of the GH-secreting PitNET samples.

Using the normalized data, a heatmap was generated, displaying the 30 genes with the most significant differential expression across the nine analyzed samples, considering their respective derivation slide and outcome (Figure 1C). In addition, an additional heatmap was created to illustrate the degree of similarity or difference between the samples (Figure 1D). Both heatmaps confirmed expression heterogeneity, regardless of whether samples were derived from recurrent or non-recurrent tumors. The Principal Component Analysis (PCA), conducted on the same normalized data, echoed these findings (Figure 1E). On PC1 (variance of 23%), only one non-recurrent sample was separated from the remaining. In comparison, samples were divided into two groups on PC2 (variance of 16%), again independently of the sample condition.

### 3.2. The lncRNAs in GH-Secreting PitNETs

The LCM-RNAseq highlighted, among the thirty common most expressed genes, a well-characterized tumor-associated lncRNA, MALAT1 (Figure 1C). MALAT1 was indeed highly expressed in both specimens derived from patient E, characterized by a combination of aggressive parameters, including very high Ki-67 expression and high Knosp grade, suggesting that these factors may contribute to the distinct transcriptomic profile.

To investigate the expression of MALAT1 and other tumor-associated lncRNAs (such as NEAT1, MEG3, and H19) with a sensitive technique, ddPCR was performed on the RNA extracted from the FFPE tissue samples of the original cohort plus six independent samples derived from PitNETs patients with overlapping clinical–pathological features. The ddPCR showed that MALAT1 is highly expressed in all samples, while H19 is almost undetectable (Table 1). Of interest NEAT1, which belongs to the same genetic locus as MALAT1, was also expressed at intermediate levels in all samples. Similar results were obtained with MEG3, which has already been reported to be one of the most significantly upregulated differentially expressed genes in somatotroph adenomas compared with other PitNET subtypes [31].

## 4. Discussion

While significant progress has been made in understanding the biology of GH-secreting PitNETs, a critical gap remains in connecting genetic alterations to specific clinical features of patients. This lack of understanding significantly hinders the development of effective and personalized treatment strategies for acromegaly, the clinical syndrome caused by excess GH production. This study aimed to address this gap by delving into the molecular landscape of GH-secreting PitNETs, focusing on unraveling the complexities of intratumoral heterogeneity.

By employing a customized approach involving LCM-RNAseq, we were able to isolate and analyze the gene expression profiles of small, homogenous cell populations within seemingly identical GH-secreting PitNET tissues. We know the number of genes/samples obtained is relatively low but sufficient to allow complete transcriptomics despite the paraffin being over 10 years old. The bioinformatic analysis highlighted the inherent heterogeneity of virtually each sample. These data are intriguing since our cohort is composed of all females. While this fact might create a bias linked to a potential sex-linked transcriptome, it strengthens, at the same time, the intrinsic diversity of a single PitNET. Indeed, recent studies reported similar findings using the spatial transcriptomic approach in a different subtype of PitNETs, pituitary corticotroph tumors. Specifically, they observed evidence of intratumor heterogeneity not based on central vs. peripheral areas but rather in clusters of diffuse cell populations within the tumor [32]. Overall, our analysis uncovered an unexpected level of molecular divergence across different samples from the same patient, emphasizing the limitations of traditional bulk tissue analysis and underscoring the importance of considering intratumoral heterogeneity in future research endeavors.

The next step in our exploration involved investigating the potential role of lncRNAs in GH-secreting PitNETs development and progression [33,34]. Growing evidence suggests that these enigmatic molecules, while not directly coding for proteins, play crucial roles in regulating gene expression and influencing various cellular processes in pituitary tumors [35,36]. Our analysis showed low expression of LINC00174 across GH-secreting PitNET samples, which contributed to the development of a variety of human cancers, exhibiting mostly oncogenic properties [37]. Conversely, we consistently observed high expression of MALAT1 and NEAT1, two lncRNAs encoded by the same genomic locus. This intriguing finding warrants further investigation into their specific contributions to GH-secreting PitNETs’ pathogenesis. In line with this, the detection of lncRNAs, including MALAT1, in liquid biopsies of PitNEt patients (e.g., serum or plasma) would be of great interest for the improvement of its significance.

Notably, we identified in our bioinformatic database some MALAT1 transcriptional targets whose function span among various cellular processes, including metabolism (PDK1), angiogenesis (VEGFA), cell adhesion (ITGB4), and stress response (NRF2). These preliminary data suggest that MALAT1 may play multifaceted roles in PitNET biology.

Despite the valuable insights gained through this exploratory study, limitations undoubtedly exist. The relatively small sample size acknowledges the need for larger-scale studies to confirm and generalize our findings, including male and female subjects. We also used FFPE samples stored for more than 10 years, which might reduce sample integrity through degradation. Therefore, validation on more recent FFPE blocks will be of great interest. Moreover, the lack of independent validation studies restricts our ability to conclusively establish the clinical relevance of the identified molecular markers. However, these limitations underscore the critical need for continued research in this domain, utilizing larger cohorts and incorporating validation steps to translate these initial findings into meaningful clinical applications.

In conclusion, this study sheds light on the intricate and heterogeneous molecular landscape of GH-secreting PitNETs, emphasizing the importance of considering intratumoral variability in future research and treatment strategies. By unveiling the diverse gene expression profiles within seemingly uniform tumors, we have laid the groundwork for a more nuanced understanding of the complex mechanisms underlying acromegaly. Our findings further highlight the potential of lncRNAs, specifically for MALAT1, which emerged directly from RNAseq and validated by ddPCR, as promising diagnostic and therapeutic targets and suggest novel avenues for developing personalized treatment approaches for patients with this challenging condition. Perspectively, larger-scale and well-designed studies are crucial to validate these findings and to translate them into meaningful clinical advancements, ultimately improving the lives of patients affected by acromegaly.

## Figures and Tables

**Table 1:** Evaluation of MALAT1, H19, NEAT1, and MEG3 transcripts by ddPCR in FFPE tissue samples.

	MALAT1	NEAT1	MEG3	H19
Patient	MEAN ± SD.	MEAN ± SD.	MEAN ± SD.	MEAN ± SD.
**A**	41,327.45 ± 7845.48	631.04 ± 120.14	242.14 ± 82.49	1.12 ± 0.58
**B**	60,690.38 ± 1508.89	299.37 ± 61.83	221.65 ± 88.91	0.41 ± 0.28
**C**	62,887.38 ± 15,820.38	745.55 ± 169.56	160.94 ± 40.72	1.99 ± 0.79
**D**	24,357.52 ± 3778.03	744.11 ± 151.57	175.66 ± 22.30	4.11 ± 1.18
**E**	131,203.57 ± 97,292.85	2733.41 ± 1732.73	606.04 ± 392.20	1.92 ± 1.64
**F**	266,572.33 ± 30,774.71	2450.00 ± 310.98	6091.61 ± 835.53	2.82 ± 0.93
**G**	267,926.36 ± 33,573.87	2222.51 ± 334.64	1855.07 ± 191.66	5.79 ± 3.39
**H**	8164.74 ± 652.60	991.11 ± 147.02	473.25 ± 30.93	1.48 ± 0.26
**I**	149,113.10 ± 5143.36	2030.48 ± 84.36	1876.73 ± 215.19	5.86 ± 1.98
**L**	247,521.69 ± 109,943.04	2345.05 ± 851.72	1810.35 ± 685.98	3.54 ± 3.25
**M**	68,399.03 ± 22,877.46	379.42 ± 103.98	213.82 ± 60.33	0.67 ± 0.35

## Data Availability

The datasets used and/or analyzed during the current study are available from the corresponding author on reasonable request.

## Supplementary Materials

The following supporting information can be downloaded at: www.mdpi.com/article/10.3390/genes15111426/s1. Figure S1: H&E staining and immunohistochemistry (IHC) decoration of GH-secreting PitNET. Representative images from patient E. IHC staining of p53, Ki67 and Synaptophysin (SYP) in PA sections, obtained through the whole slide imaging (WSI) using NanoZoomer 2.ORS at 20× magnification. Figure S2: Schematic of Laser Microdissection. (A) H&E staining of GH-secreting PitNET. Blue line = selected area (ROI = region of Interest) corresponding to about 50 cells; (B) Tissue section as in A on polyphenylene sulfide membrane (PPS) for LCM dissection. Green line = c utting perimeter; Red circle = recovered area; (C) tissue cutting along the green line; (D) Tissue after cutting; (E) cut ROI. Area were microdissected on different caps and processed separately for RNA-seq. Scale bar is indicated. Figure S3: Bar-plot of expressed genes plotted as reads count in each sample. Of note, sample Pit29 was obtained from 2 LCM areas in the same tube. Figure S4: Hematoxylin and Eosin (H&E) staining of GH-secreting PitNET sections. Representative H&E stained sections obtained through the whole slide imaging (WSI) using NanoZoomer 2.ORS at 20× magnification. (A) Patient A. (B) Patient B. (C) Patient C. (D) Patient D (E) Patient E. Table S1. Clinical and pathological data of GH-secreting PitNET cohort. Table S2. Recovered sample areas upon LCM and relative data. Table S3. Genes in common between samples from the same slide.
