# Supplementary material for: LCM-RNAseq Highlights Intratumor Heterogeneity and a lncRNA Signature from Archival Tissues of GH-Secreting PitNETs
Source: Genes (Basel). 2024 Oct 31;15(11):1426. doi: 10.3390/genes15111426 (PMC11593583; doi:10.3390/genes15111426)
Supplement: Supplementary file 1 [file genes-15-01426-s001.zip › genes-3225526-supplementary.pdf]

Table S1. Clinical and pathological data of GH-secreting PitNET cohort

Patient	Sample	Age	Gender	GH (ng/ml)	IGF-I (ng/ml)	Histology	Cytokeratin expression pattern	Max Diameter (mm)	Knosp grade	Ki67	Recurrence
A	Pit 4<br>Pit 3	60	F	6,2	666	GH secreting macro-adenoma	Densely granulated	27	1	<1%	Yes
B	Pit 11	59	F	7,2	601	GH secreting macro-adenoma	Densely granulated	21	1	<1%	No
C	Pit 34<br>Pit 35<br>Pit 36	28	F	8,5	654	GH secreting macro-adenoma	Sparsely granulated	24	1	3%	No
D	Pit 1	25	F	9,2	727	GH secreting macro-adenoma	Sparsely granulated	16	0	1%	No
E	Pit 18<br>Pit 29	24	F	15	1200	GH secreting macro-adenoma)	Sparsely granulated	43	4	6-7%	Yes

Table S2. Recovered sample areas upon LCM and relative data

Patient	Outcome	Samples	Sampling area (µm²)	Genes with ≥10 reads
A	Recurrent	Pit3	13700	150
		Pit4	13500	306
B	Non-Recurrent	Pit11	14200	50
C	Non-Recurrent	Pit34	14400	413
		Pit35	15000	683
		Pit36	14000	150
D	Non-Recurrent	Pit1	15000	599
E	Recurrent	Pit18	14800	83
		Pit29	14000+17000	2561

Table S3: Genes in common between samples from the same slide

Patient	Sample	Common genes
A	Pit3, Pit4	**RN7SL2**, TEX101
B	Pit11	/
C	Pit34, Pit35, Pit36	RNA5S9, RNA5SP149, **RN7SL1**, TEX101
D	Pit1	/
E	Pit18, Pit29	ARHGAP29, CSDE1, PDE4DIP, RGS2, RNA5S9, TNS1, **ARHGAP31**, RNA5SP145, RNA5SP149, YEATS2, IGF2R, MP3K4, ACTB, ACOT9, FBXO32, MAPK8, EIF3F, NEAT1**, MALAT1**, ARF3, RPPH1, **RN7SL1, RN7SL2**, RNA5SP429, STAT3, EPG5, CIRBP, **MT-CO1**

Genes in bold are present in the Heatmap of Figure 1C

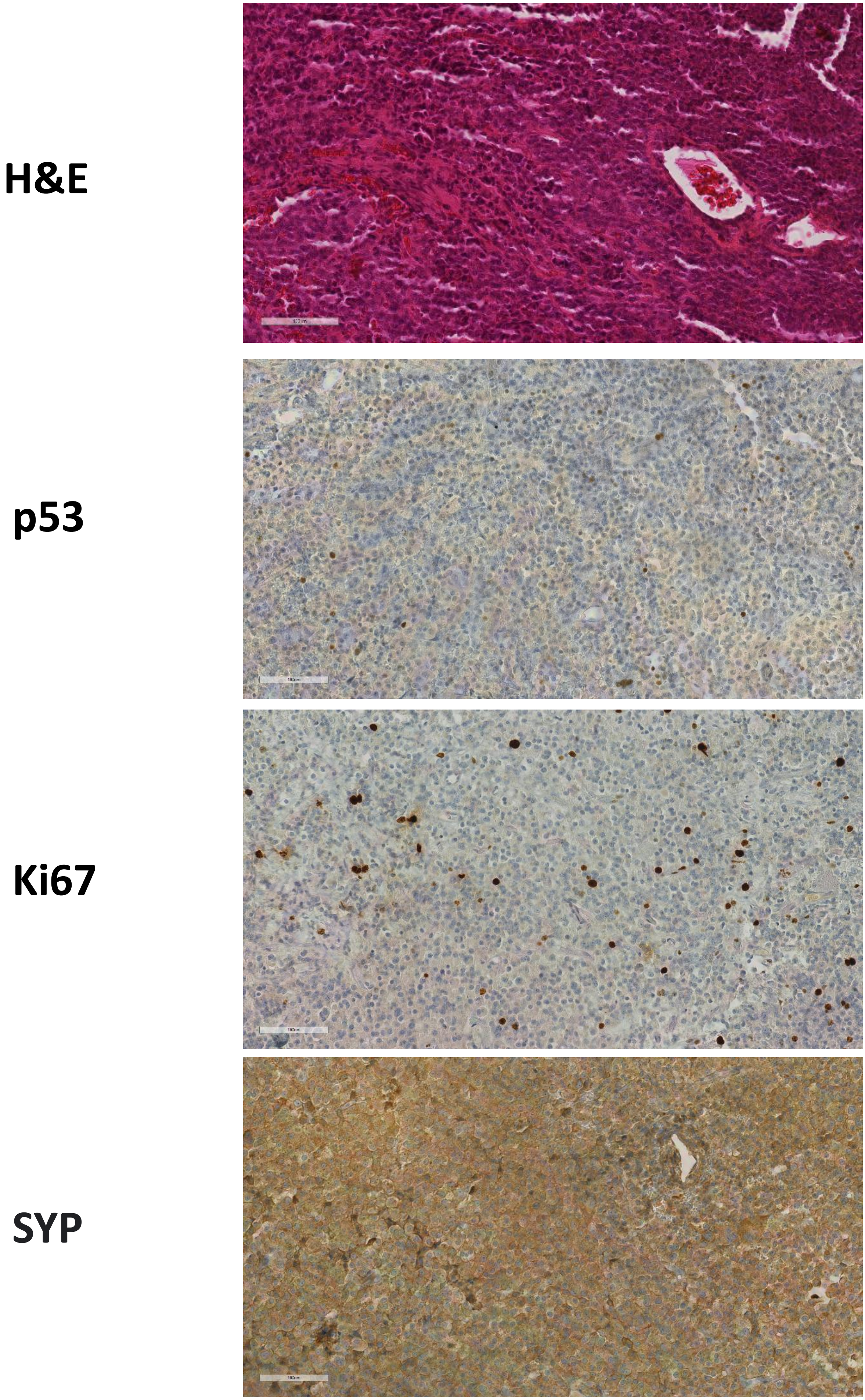

Figure S1: H&E staining and immunohistochemistry (IHC) decoration of GH-secreting PitNET. Representative images from patient E. IHC staining of p53, Ki67 and Synaptophysin (SYP) in PA sections, obtained through the whole slide imaging (WSI) using NanoZoomer 2.ORS at 20x magnification.

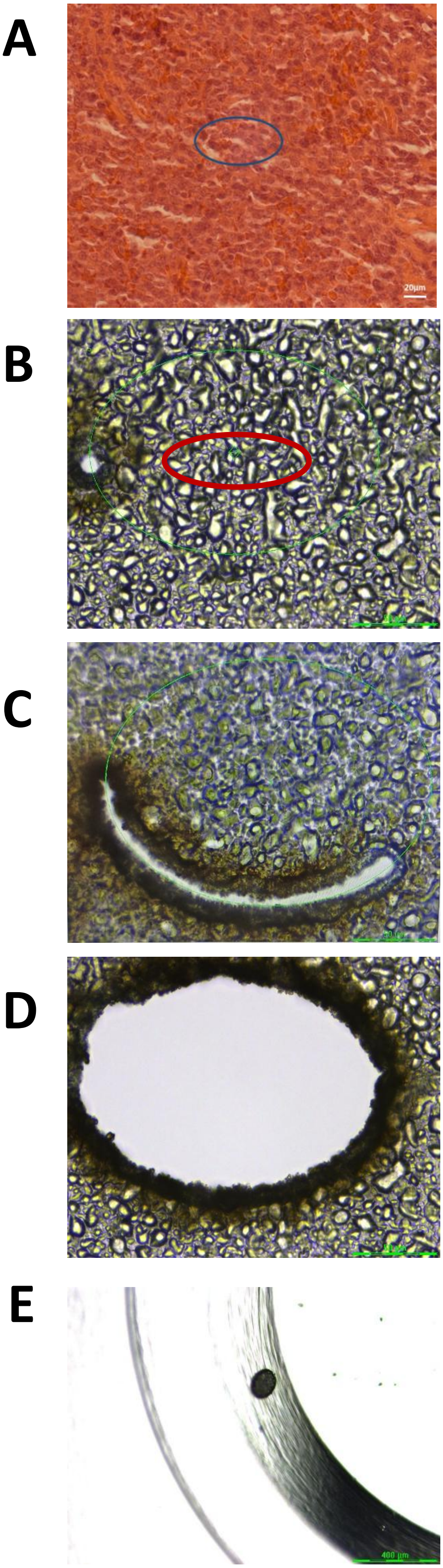

Figure S2: Schematic of Laser Microdissection. (A) H&E staining of GH-secreting PitNET. Blue line= selected area (ROI=region of Interest) corresponding to about 50 cells; (B) Tissue section as in A on polyphenylene sulfide membrane (PPS) for LCM dissection. Green line =cutting perimeter; Red circle= recovered area; (C) tissue cutting along the green line; (D) Tissue after cutting; (E) cut ROI. Area were microdissected on different caps and processed separately for RNA-seq. Scale bar is indicated

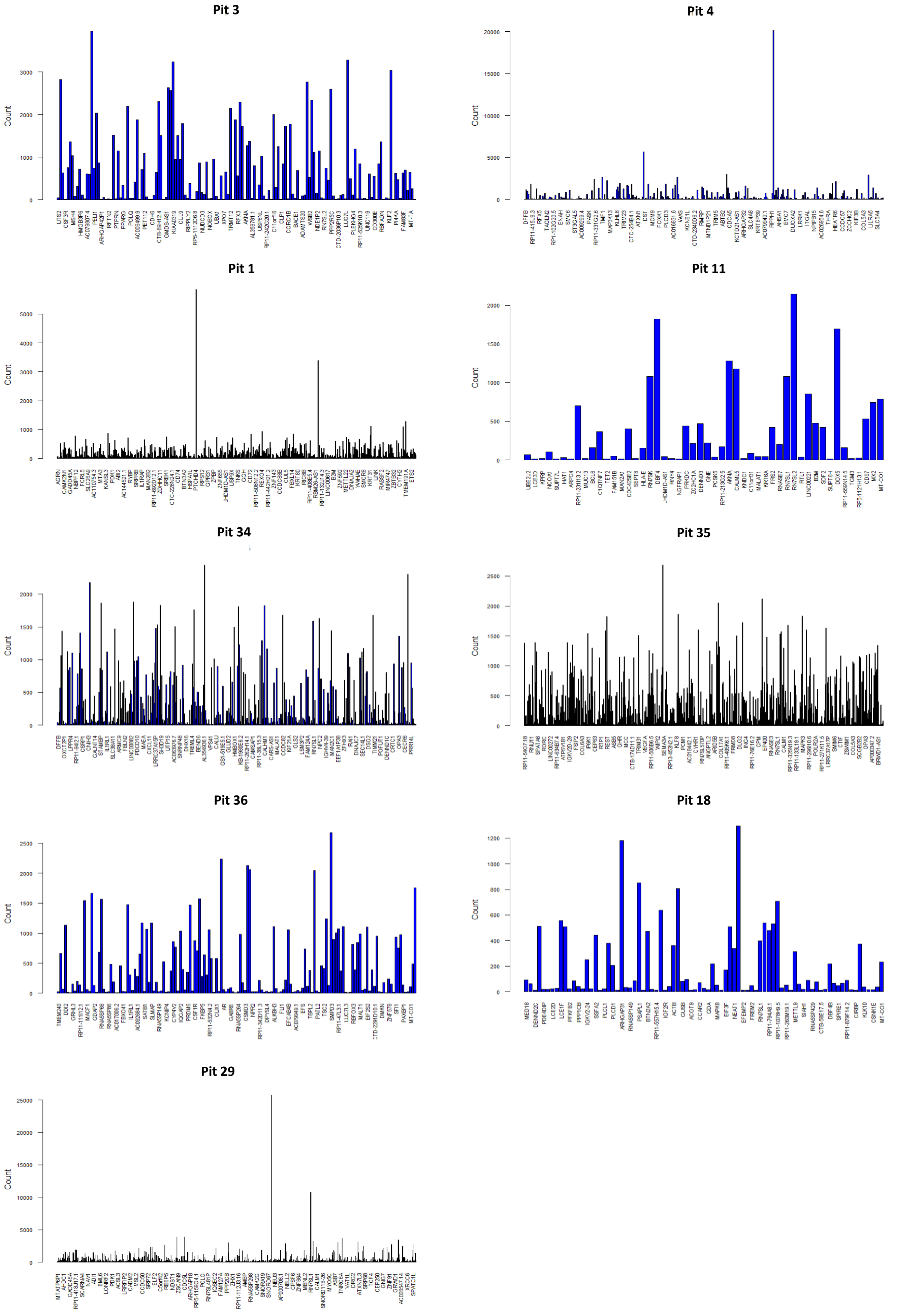

Figure S3: Bar-plot of expressed genes plotted as reads count in each sample. Of note, sample Pit29 was obtained from 2 LCM areas in the same tube

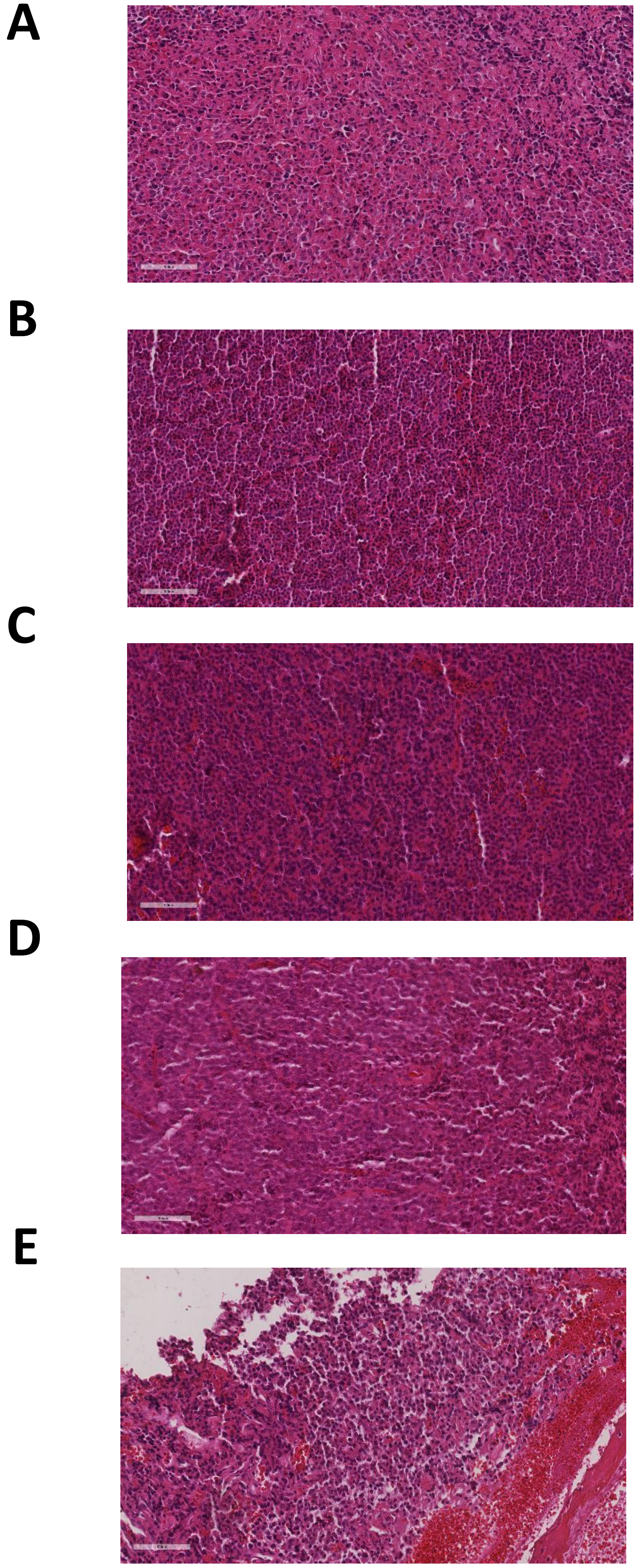

Figure S4: Hematoxylin and Eosin (H&E) staining of GH-secreting PitNET sections. Representative H&E stained sections obtained through the whole slide imaging (WSI) using NanoZoomer 2.ORS at 20x magnification.(A) Patient A. (B) Patient B. (C) Patient C. (D) Patient D (E) Patient E
